# A brief period of sleep deprivation leads to subtle changes in mouse gut microbiota

**DOI:** 10.1111/jsr.12920

**Published:** 2019-09-12

**Authors:** Sahar El Aidy, Youri G. Bolsius, Frank Raven, Robbert Havekes

**Affiliations:** ^1^ Department of Molecular Immunology and Microbiology Groningen Biomolecular Sciences and Biotechnology Institute (GBB) University of Groningen Groningen The Netherlands; ^2^ Groningen Institute for Evolutionary Life Sciences (GELIFES) University of Groningen Groningen The Netherlands

**Keywords:** gut, mice, microbiome, microbiota, sleep, sleep deprivation

## Abstract

Not getting enough sleep is a common problem in our society and contributes to numerous health problems, including high blood pressure, diabetes and obesity. Related to these observations, a wealth of studies has underscored the negative impact of both acute and chronic sleep deprivation on cognitive function. More recently it has become apparent that the gut microbiota composition can be rapidly altered, modulates brain function and is affected by the aforementioned health problems. As such, changes in the microbiota composition may contribute to the behavioural and physiological phenotypes associated with sleep deprivation. It is unclear, however, whether a brief period of sleep deprivation can also negatively impact the gut microbiota. Here, we examined the impact of 5 hr of sleep deprivation on gut microbiota composition of male C57Bl6/J mice. Despite the fact that the overall microbial composition did not change between the control‐ and sleep‐deprived groups, the relative abundance of the *Clostridiaceae* and *Lachnospiraceae* were slightly altered in sleep‐deprived animals compared to controls. Together, these data suggest that depriving mice of sleep for 5 hr leads to subtle changes in the gut microbiota composition.

## 1 INTRODUCTION

All mammals, including humans, spend a large proportion of their lives asleep, time that is not spent on finding a partner, searching for nutrients or detecting danger. As such, one can only conclude that sleep has maintained its biological relevance across time. Despite the underscored importance of sleep, our sleep time has been reduced from approximately 8 to 6.5 hr per night (Survey 2005). This reduction has been reported to be a result of many factors, including working longer and increased social demands, as well as the more frequent usage of electronic devices in bed such as mobile phones, laptops, etc. The resulting deprivation and restriction of sleep impacts our body in many ways. Indeed, numerous studies have underscored the negative impact of sleep loss on our well‐being (Bryant, Trinder, & Curtis, 2004; Harrison, Horne, & Rothwell, 2000). For example, even as little as 5 hr of sleep deprivation negatively impacts brain function through the misregulation of numerous signalling pathways (Abel, Havekes, Saletin, & Walker, 2013; Havekes & Abel, 2017; Havekes, Meerlo, & Abel, 2015; Havekes, Vecsey, & Abel, 2012; Puentes‐Mestril & Aton, 2017; Raven, Van der Zee, Meerlo, & Havekes, 2018). A wealth of studies have emphasized that the hippocampus, a brain region critical for learning and memory formation, is particularly sensitive to sleep loss (Abel et al., 2013; Bjorness, Riley, Tysor, & Poe, 2005; Delorme, Kodoth, & Aton, 2019; Hagewoud, Bultsma, Barf, Koolhaas, & Meerlo, 2011; Hagewoud, Havekes, Novati, et al., 2010; Havekes & Abel, 2017; Havekes et al., 2015; McCoy et al., 2013; Mueller, Meerlo, McGinty, & Mistlberger, 2015; Prince et al., 2014). Likewise, more chronic sleep restriction lasting days to weeks leads to misregulation of synaptic plasticity and even hippocampal shrinkage in rodents (Kreutzmann, Havekes, Abel, & Meerlo, 2015; Meerlo, Mistlberger, Jacobs, Heller, & McGinty, 2009; Meerlo, Sgoifo, & Suchecki, 2008; Novati, Hulshof, Koolhaas, Lucassen, & Meerlo, 2011). The latter observation is in line with human studies suggesting that sleep apnea and in some cases insomnia can even lead to a reduction in hippocampal volume (Morrell et al., 2010; Riemann et al., 2007). Altogether, these studies emphasize that sleep deprivation can negatively impact brain function.

Beyond affecting specific signalling pathways and regions in the brain, chronic loss of sleep can also indirectly affect brain function through the modulation of peripheral signalling mechanisms. More recently, it has become apparent that the gut microbiota composition is highly plastic, can be abruptly altered, and can even modify behaviours such as eating behaviour, sexual behaviour, social behaviour, cognition and addiction (Cussotto, Sandhu, Dinan, & Cryan, 2018). Indeed, the gut microbiota has been implicated in a variety of conditions, including anxiety, depression and neurodegenerative disorders, although this work is largely based on animal studies or association studies in patient populations (Koopman & El Aidy, 2017; Sylvia & Demas, 2018; Waclawikova & El Aidy, 2018; Warner, 2019). The impact of sleep deprivation and sleep restriction on the gut microbiome, however, remains unclear. For example, Poroyko and colleagues showed that 4 weeks of chronic sleep fragmentation alters the gut microbiota in mice (Poroyko et al., 2016). Likewise, Anderson et al. (2017) reported a possible relationship between sleep quality and the composition of the human gut microbiome (Anderson et al., 2017). In contrast, recent work by Zhang and colleagues (Zhang et al., 2017) suggested that chronic sleep restriction does not lead to gross changes in the faecal microbiota of either humans or rats. The discrepancy between the rodent studies suggests that the microbiota composition may be more susceptible to long‐term changes in sleep quality rather than sleep quantity acutely, as noted by Zhang et al. (2017). Despite the observation that more chronic sleep fragmentation can negatively impact the gut microbiota, it is unclear whether a single brief period of sleep deprivation could impact the composition of gut microbiota in addition to affecting cognitive processes. The latter becomes even more interesting as even short‐term exposure to stress has been shown to alter the microbiota community profile (Galley et al., 2014). For this reason, in the current paper we examined the impact of a single 5‐hr period of sleep deprivation on the gut microbiota of adult mice.

## 2 METHODS

### 2.1 Mice

Male C57BL/6J mice were obtained from Charles River Laboratories (Sulzfeld, Germany) at an age of 6 weeks, housed in random pairs upon delivery and kept in the same room under specific pathogen free conditions. Animals were housed on a 12‐hr light 12‐hr dark schedule with lights on at 07:00 hours (ZT 0). Mice had food and water available ad libitum. At an age of 9 weeks, 1 week prior to the sleep deprivation experiment, pairs were separated and one animal from each pair was randomly assigned to the sleep deprivation group and one to the non‐sleep‐deprivation control group. In this way, cage effects could be excluded. Experimental protocols were reviewed and approved by the Dutch Committee for Animal Experiments (CCD) in accordance with European laws for animal research and the Animal Care and Use Committee of the University of Groningen.

### 2.2 Sleep deprivation

During the habituation phase, all mice were handled for 2 min on 5 consecutive days prior to the sleep deprivation (SD) experiment in the experimental room in order to habituate them to both experimenters without affecting synaptic plasticity (Vecsey et al., 2013). This was also done to prevent any contamination specifically in the batch of SD mice. After the habituation, the mice were randomly assigned to the control (C) or SD group. The animals of the SD group were sleep deprived for 5 hr, starting at lights out (ZT0). SD was achieved by the gentle stimulation method as described in our previously published papers (Hagewoud, Havekes, Novati, et al., 2010; Hagewoud, Havekes, Tiba, et al., 2010; Havekes et al., 2014, 2016; Raven, Meerlo, Van der Zee, Abel, & Havekes, 2019; Tudor et al., 2016). Briefly, animals were kept awake by gently tapping the cage, gently shaking the cage and/or removing the wire cage top. Their bedding was disturbed only in cases when tapping or gently shaking the cage was insufficient. Importantly, we did not use any novel objects, cages or other arousing stimuli to keep the animals awake. We were also aware that additional food intake could occur in the mice that were kept awake for 5 hr, which may impact the microbiome (Daniel et al., 2014; Thaiss et al., 2014). However, food restriction by giving animals a cage with novel bedding will have a major impact on sleep quality and duration as well as physiology in both the control and sleep‐deprived animals.

This method of SD was previously validated using electroencephalogram (EEG) recordings and prevents all rapid eye movement (REM) sleep and approximately 95% of all non‐rapid eye movement (NREM) sleep (Meerlo, de Bruin, Strijkstra, & Daan, 2001). Furthermore, previous work by us and others has indicated that the deficits in memory and plasticity as a result of SD were not caused by elevated plasma corticosterone levels or the gentle stimulation method itself (van der Borght et al., 2006; Meerlo & Turek, 2001). The role of glucocorticoids in the endophenotypes related to SD has been extensively discussed previously (Havekes et al., 2012, 2015; Kreutzmann et al., 2015).

### 2.3 Colonic samples processing and microbiota profiling

The luminal content of the colon was collected from the control group and SD group directly after their exposure to 5 hr of sleep deprivation. Samples were snap frozen and stored at −80**°**C until further processing. DNA was extracted according to the manufacturer's instructions using the NucleoSpin^®^ Soil Kit (Macherey Nagel, GmbH & Co). High‐throughput sequencing of the V3‐V5 hypervariable region of the bacterial 16S rRNA gene was performed by Eurofins on an Illumina MiSeq platform according to the standard protocols with minor adjustments. Briefly, the V3‐V5 region was PCR amplified using universal primers that contained the adapter overhang nucleotide sequences for forward and reverse index primers. Primers used are 16S V3‐V5 *Fwd* CCTACGGGNGGCWGCAG (Klindworth et al., 2013) and 16S V3‐V5 *Rev* GGGTTGCGCTCGTTGCGGG (Sacchi et al., 2002). Amplicons were purified using AMPure XP beads (Beckman Coulter) and set up for the index PCR with Nextera XT index primers (Illumina). The indexed samples were purified using AMPure XP beads, quantified using the Fragment Analyzer (Advanced Analytical) and equal quantities from each sample were pooled. The resulting pooled library was quantified using the Bioanalyzer 7500 DNA Kit (Agilent) and sequenced using the v3 chemistry (2 × 300 bp paired‐end reads).

### 2.4 Bioinformatics and statistical analyses

Microbial ecology (QIIME) software was used and preliminary quality control steps were performed as described previously (Kuczynski et al., 2012) and chimera sequences were removed with ChimeraSlayer. The remaining effective sequences were binned into operational taxonomic units (OTUs) with a cut‐off of 97% identity to determine alpha diversity (Shannon, Simpson and evenness indices), richness abundance‐based coverage estimator (ACE and Chao1) and Good's coverage. Beta diversity was estimated by computing weighted UniFrac values and visualized using a principal coordinate analysis (PCoA). Comparisons among groups were performed using the adonis function (ANOVA using distance matrices) of the permutational multivariate ANOVA (PERMANOVA) implemented in the Vegan R package. Samples with insufficient read counts were filtered. Samples with less than 1,000 sequence reads were removed. A linear discriminant analysis effect size (LEfSe) procedure was used to identify the biomarkers of the microbiota in the colonic samples at the family and genus levels, and an effect size threshold of 2 (on the logarithmic linear discriminant analysis [LDA] score) was used (Segata et al., 2011). Compared with standard statistical approaches, LEfSe, in addition to *p*‐values, provides an estimate of the magnitude of the association between each OTU and the grouping categories based on the LDA score. All statistical analyses were performed using the SPSS data analysis program (version 21.0) and R packages (V.2.15.3). *p*‐values < .05 were considered to indicate statistical significance.

## 3 RESULTS

### 3.1 Acute sleep deprivation does not affect the diversity of the gut microbiota

**Figure 1:**
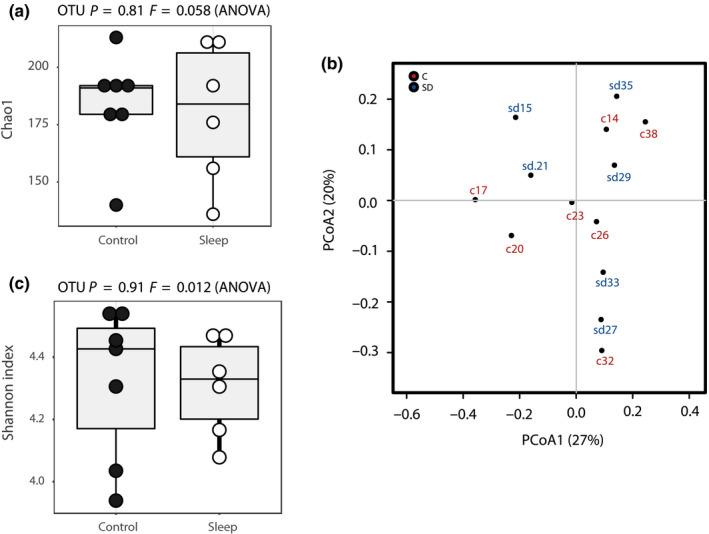
(a) Comparison of Shannon index between sleep deprivation (SD) and control (C) groups (*p* = .91 *F* = 0.012, ANOVA) and of Chao index between SD and C groups (*p* = .81 *F* = 0.058, ANOVA). **(**b**)** Principal coordinates analysis (PCoA) based on weighted UniFrac analysis between SD and C groups

To investigate the differences between the colonic luminal microbial communities in SD and control groups, the ecological characteristics were evaluated using various indices based on the OTU level. Compared to the control group, the SD group exhibited similar Shannon, ACE and Chao indices (Figure 1a). To identify the between‐subject similarity of microbial communities, a weighted UniFrac‐based principal coordinated analysis (PCoA) was performed. PCoA of control versus SD groups revealed that the overall microbial composition did not deviate between the tested groups (PERMANOVAR, *p *=* *.955, *R* ^2^ = .05; Figure 1b).

### 3.2 Differentially abundant bacterial groups in mice following acute sleep deprivation

**Figure 2:**
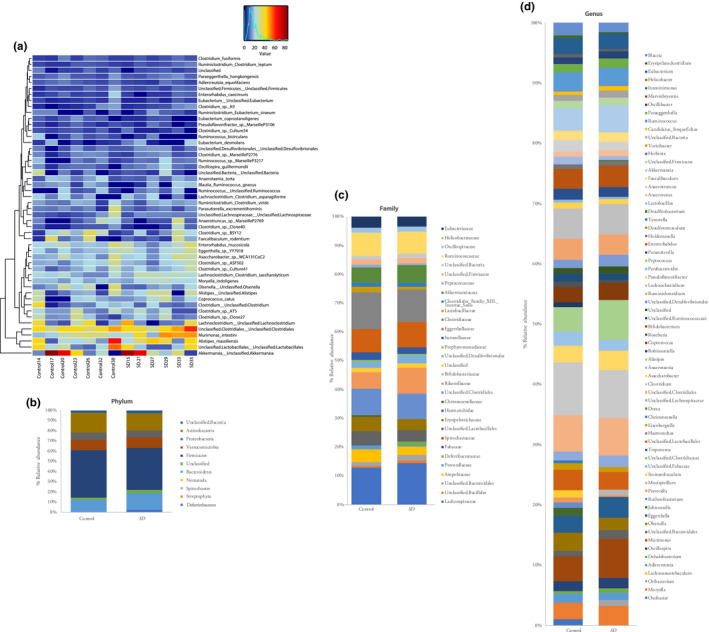
(a) Heatmap showing quantitative visualization of microbial community composition. Rows of the generated heatmap represent taxa; columns represent samples. Both are ordered by hierarchical clustering. Taxa abundances are presented in a colour code, ranging from red (highly abundant) to blue (rare or absent). Values of explanatory variables are presented as a separate heatmap on top of the main heatmap. Stacked bar graphs showing altered gut microbiota composition on (B) phylum, (c) family and (d) genus levels, respectively

Next, we examined whether groups of OTUs with commonly assigned taxonomic ranks of the faecal microbiota were changed by acute sleep restriction (Figure 2a). No changes were detected at the phylum level (Figure 2b). At the family level, Lachnospiraceae has a tendency to be more abundant (*p = *.035, false discovery rate [*FDR *] = 1) in the SD group compared to the control group (Figure 2c). At the genus level, Oxobacter was more prominently present (*p =* .0066, *FDR* = 0.44) in the control group, whereas Moryella was more abundant (*p =* .022, *FDR* = 0.74) in the SD group (Figure 2d). At the species level, *Oxobacter PPf50E4* (*p* = .0066, *FDR* = 0.81) was more enriched in the control group, whereas *Moryella indoligenes* (*p = 0.022*,*FDR = 1*) and *Clostridium saccharolyticum* (*p* = .03, *FDR* = 1) were more abundant in the SD group. Among the groups of OTUs with commonly assigned taxonomic ranks, despite the mildly significant *p*‐values, none of the OTUs were statistically different between the microbiota of control mice and that of acute sleep‐restricted mice based on the FDRs. Of these OTUs, *Murimonas intestine_00000017* (*p* = .035, *FDR* = 1), *Murimonas intestine_00000298* (*p = *.05, *FDR* = 1) and *Oscillospira guilliermondii__00000235* (*p* = .05, *FDR* = 1) had a tendency to be higher in the faecal samples of SD mice. In contrast, *Clostridiales_00000238* (*p *= .042, *FDR *= 1), *Oxobacter__s__sp._PPf50E4_00000036* (*p *= .045, *FDR *= 1) and *Clostridiales Clostridiales_00000255* (*p =* .045, *FDR *= 1) were more abundant in control mice (Table S1).

**Figure 3:**
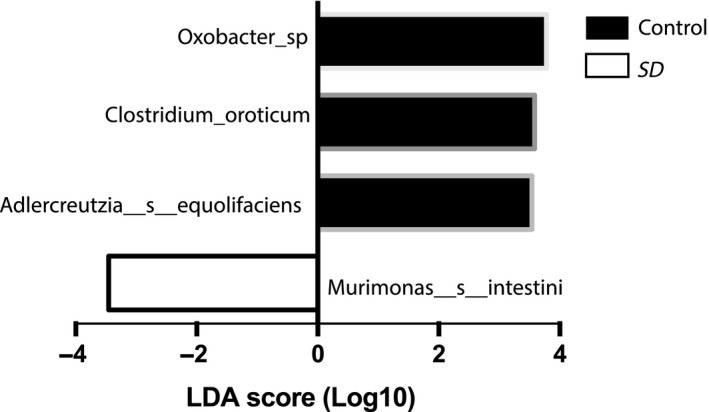
A linear discriminant analysis (LDA) effect size (LEfSe) identifying microbial biomarkers at the operational taxonomic unit (OTU) level that discriminates between control and sleep deprivation (SD) groups of mice

Finally, we used LDA LEfSe to identify the taxa most likely to explain differences in the microbiota composition between control and SD animals. When comparing both groups of mice, we found that three species (*Oxobacter PPf50E4*,*Clostridium oroticum*, and *Adlercreutzia equolifaciens*) were more abundant in the control group, whereas *Murimonas intestini* was over‐represented in the SD group (Figure 3). Together, these findings suggest that the gut microbiota exhibit very subtle, if any, changes in their composition following a brief period of sleep deprivation.

## 4 DISCUSSION

In the present study, we showed that a single short period of SD in mice does not cause major shifts in the gut microbiota composition, but leads to subtle changes in the differential abundance of microbiota on specific taxa levels. These include members of the family Lachnospiraceae and members of the family Clostridiaceae, which belong to the Firmicutes phylum. This is in agreement with the study of Benedict et al. (2016), who reported an increase in the Firmicutes:Bacteroidetes ratio following two nights of restricted sleep versus normal sleep in humans (Benedict et al., 2016). Unlike our observation, Benedict et al. (2016) detected higher abundances of the families Coriobacteriaceae and Erysipelotrichaceae, and lower abundance of Tenericutes. This discrepancy could be due to differences in model organisms used (mice versus humans) and the targeted variable regions that were used for microbiota composition analysis (V3‐V5 versus V4). Another study examining chronic sleep restriction in rats and humans, demonstrated that changes in the composition of the microbiota were remarkably resistant to sleep deprivation across species (Zhang et al., 2017). Zhang et al. (2017) targeted V1‐V2 hypervariable regions for DNA extraction from faecal samples and applied a cross‐species approach in both rat and human studies of sleep restriction. It is also important to note that Zhang et al. (2017) applied chronic sleep restriction using rotating drums (7 days, 20 hr/day), whereas in our studies we used the gentle handling method (1 day, 5 hr) to deprive animals of sleep in line with our previous work. These differences observed between the two studies might also be attributed to the difference in timing and method of sampling. In our acute SD experiment, colonic content was freshly collected at ZT5; however, in the chronic SD study, samples were collected at ZT8‐12. It is very important to consider the timing of sample collection when comparing studies of the gut microbiota as evidence for oscillation of these microbes and for a bidirectional interaction between their rhythm and the host circadian clock has now been well established (Montagner et al., 2016). Moreover, additional food intake could occur in the mice that were kept awake for 5 hr in our study, which may impact the microbiome (Daniel et al., 2014; Thaiss et al., 2014). However, food restriction by giving animals a cage with novel bedding will have a major impact on sleep quality and duration as well as physiology in both the control and sleep‐deprived animals.

Notably, intestinal inflammation is well known to impact the gut microbiota and studies have found that intestinal inflammation can also reduce the abundance of the genus *Oxobacter* (Rigsbee et al., 2012; Shankar, Agans, Holmes, Raymer, & Paliy, 2013), the species *C. oroticum* (Du et al., 2015) and *Adlercreutzia equolifaciens* (Shaw et al., 2016). However, Galley et al. (2014) did not report any significant changes in inflammatory cytokine gene expression after exposure to a murine stressor for 2 hr, for either 1 or 6 consecutive days (Galley et al., 2014). This observation makes it highly unlikely that acute sleep deprivation results in stressor‐induced intestinal inflammation, which could then ultimately lead to alteration in the abundances of the indigenous intestinal microbiota. Indeed, in our previous sleep deprivation studies we have assessed the impact of sleep deprivation on HPA‐axis activation and showed that 5–6 hr of sleep deprivation leads to a minor increase in blood corticosterone levels comparable to the fluctuations observed across the 24‐hr circadian cycle (van der Borght et al., 2006; Hagewoud, Havekes, Novati, et al., 2010; Palchykova, Winsky‐Sommerer, Meerlo, Durr, & Tobler, 2006; Vecsey et al., 2013). The role of glucocorticoids in sleep deprivation‐induced phenotypes has been discussed in our previous work (Havekes et al., 2012, 2015).

Intriguingly, in line with the subtle changes in the microbiota composition we observed following acute SD, recent work has reported similar alterations following short‐term exposure to stress in mice (Galley et al., 2014). For example, in our study, SD mice had increased levels of *Lachnospiraceae*. Members of the family *Lachnospiraceae* within the order *Clostridiales* represent one of the most dominant and prevalent bacterial groups within the *Firmicutes*. Changes in the occurrence of members of the *Lachnospiraceae* have been associated with as little as 2 hr of stressor exposure in mice (Galley et al., 2014). Despite these similarities, it should be noted that a brief period of sleep deprivation similar to the one used in this study does not lead to a robust HPA‐axis activation resulting in robustly elevated glucocorticoid levels (van der Borght et al., 2006). In fact, the glucocorticoid levels never reached levels beyond those observed across the 24‐hr circadian cycle. Furthermore, removal of the adrenal glands did not prevent SD‐induced memory impairments in a hippocampus‐dependent form of learning (Tiba, Oliveira, Rossi, Tufik, & Suchecki, 2008). The latter observation underscores the notion that SD rather than the mild arousal as a result of sleep deprivation or the sleep deprivation method underlies the widely reported impairments in hippocampal function. Finally, it is important to emphasize the fact that sleep deprivation per se leads to memory impairments, whereas slight increases in glucocorticoid levels if anything are beneficial for memory and synaptic plasticity (for extensive discussion see Havekes et al. 2015, 2012). Altogether, these data suggest that acute sleep deprivation and stress can both lead to increased levels of *Lachnospiraceae*. Based on the work described above, however, it is unlikely that such changes directly contribute to the memory deficits associated with a brief period of sleep deprivation.

The hippocampus, a region critical for learning and memory, is particularly sensitive to sleep loss (Abel et al., 2013; Delorme et al., 2019; Hagewoud, Havekes, Novati, et al., 2010; Hagewoud et al., 2011; Havekes et al., 2012, 2015; McCoy et al., 2013; Mueller et al., 2015; Prince et al., 2014; Tudor et al., 2016) and its impairment has been shown to cause abnormalities in gastrointestinal motility (Xia et al., 2016), a key regulator of microbiota composition (Vandeputte et al., 2016). For this reason, it is tempting to speculate that the changes seen at the microbiota level are a consequence of altered motility that results from the negative impact of sleep deprivation on hippocampal function. To test this hypothesis, in future studies it would be highly interesting to assess the changes in gut motility during the course of acute sleep deprivation and relate them to both changes in hippocampal function and subtle alterations in the composition of the gut microbiota.

## 5 CONCLUSION

Previous studies have found that exposure to as little as 2 hr of a social stressor is sufficient to significantly affect some populations of the gut microbiota. Here, we show that 5 hr of total sleep deprivation also leads to specific changes in the gut microbiota of male mice. Despite the subtle changes observed in the microbiota composition, we do not have evidence that specific changes seen here are affecting behaviour. Future studies will have to elucidate the molecular mechanisms that are ultimately responsible for these changes and how these changes may contribute to behavioural and neurophysiological phenotypes associated with loss of sleep.

## CONFLICT OF INTEREST

No conflicts of interest declared.
