# Supplementary material for: A brief period of sleep deprivation leads to subtle changes in mouse gut microbiota
Source: J Sleep Res. 2019 Sep 12;29(6):e12920. doi: 10.1111/jsr.12920 (PMC7757181; doi:10.1111/jsr.12920)
Supplement: Supplementary file 1 [file JSR-29-e12920-s001.pdf]

Taxa.__OTU___	P (rank test)	Adjusted P (Bonf.)	FDR	control mea	sleep mean	control median	sleep median	% Relative abundance (control)	% Relative abundance (SD)	Fold Change
p__Firmicutes__g__Murimonas__s__intestini_00000017	0,035	1	0,96	14,82	22,48	14,76	25,12	1,180856681	1,925582964	-1,517
p__Firmicutes__o__Clostridiales_00000238	0,043	1	0,96	3,73	0,67	4,9	0	0,392018817	0	5,593
p__Firmicutes__o__Clostridiales_00000255	0,045	1	0,96	3,29	0	5,57	0	0,44562139	0	13450
p__Firmicutes__g__Oxobacter__s__sp._PPf50E4_00000036	0,045	1	0,96	8,08	0	3,46	0	0,276813287	0	33000
p__Firmicutes__g__Oscillospira__s__guilliermondii_00000235	0,053	1	0,96	0	4,23	0	2,74	0	0,210035721	-17250
p__Firmicutes__g__Murimonas__s__intestini_00000298	0,053	1	0,96	0	3,65	0	3,39	0	0,259861714	-14890
p__Firmicutes__o__Clostridiales_00000114	0,066	1	0,96	2,18	7,86	0	6,52	0	0,49979303	-3,61
p__Firmicutes__g__Clostridium__s__sp._Culture41_00000174	0,066	1	0,96	1,18	4,57	0	3,81	0	0,29205697	-3,885
p__Firmicutes__o__Clostridiales_00000132	0,073	1	0,96	4	6,37	5,39	6,73	0,431220699	0,515890659	-1,593
p__Firmicutes__g__Moryella__s__indoligenes_00000048	0,099	1	0,96	8,71	12,64	10,05	12,87	0,804038594	0,986554648	-1,451
p__Firmicutes__o__Clostridiales_00000215	0,1	1	0,96	3,2	0	0	0	0	0	13050
p__Firmicutes__g__Stomatobaculum__s__longum_00000359	0,1	1	0,96	2,62	0	0	0	0	0	10690
p__Firmicutes__g__Ruminococcus__s__bicirculans_00000188	0,1	1	0,96	6,4	1,6	8,31	0	0,664831912	0	4,001
p__Firmicutes__g__Clostridium__s__sp._Culture54_00000268	0,1	1	0,96	2,28	0,55	3,74	0	0,299214362	0	4,124
p__Firmicutes__g__Clostridium__s__sp._BSY12_00000327	0,1	1	0,96	1,75	0	0	0	0	0	7138
p__Firmicutes__g__Clostridium__s__sp._BSY12_00000272	0,1	1	0,96	2,06	0	0	0	0	0	8421
p__Firmicutes__g__Clostridium_00000295	0,1	1	0,96	1,85	0	0	0	0	0	7535
p__Firmicutes__g__Eubacterium__s__coprostanoligenes_00000140	0,11	1	0,96	5,34	1,92	6,71	0	0,536825768	0	2,79
p__Firmicutes__g__Clostridium__s__sp._Culture57_00000123	0,11	1	0,96	2,69	5,22	3,61	5,34	0,288813863	0,409339691	-1,942
p__Firmicutes__g__Lachnoclostridium__s__Clostridium_oroticum_00000112	0,12	1	0,96	9,33	5,25	9,7	6,36	0,77603725	0,487528171	1,778
p__Firmicutes__g__Eubacterium__s__coprostanoligenes_00000072	0,12	1	0,96	7,93	3,27	7,87	0	0,629630222	0	2,423
p__Firmicutes__g__Clostridium__s__sp._Culture41_00000127	0,13	1	0,96	1,59	5,2	0	4,69	0	0,359513698	-3,269
p__Firmicutes__g__Clostridium__s__sp._Clone40_00000058	0,13	1	0,96	7,45	3,53	6,08	1,73	0,486423348	0,132613795	2,109
p__Firmicutes__g__Clostridium__s__sp._AT5_00000073	0,13	1	0,96	4,9	6,74	4,58	6,95	0,366417588	0,532754841	-1,377
p__Firmicutes__o__Clostridiales_00000459	0,14	1	0,96	0	1,56	0	0	0	0	-6355
p__Firmicutes__o__Clostridiales_00000404	0,14	1	0,96	0	1,72	0	0	0	0	-7021
p__Firmicutes__g__Oribacterium__s__sp._oral_taxon_078_00000169	0,14	1	0,96	2,15	6,53	0	7,83	0	0,600211569	-3,032
p__Firmicutes__g__Murimonas__s__intestini_00000425	0,14	1	0,96	0	2,13	0	0	0	0	-8695
p__Firmicutes__g__Murimonas__s__intestini_00000346	0,14	1	0,96	0	2,54	0	0	0	0	-10380
p__Firmicutes__g__Murimonas__s__intestini_00000292	0,14	1	0,96	0	2,99	0	0	0	0	-12210
p__Firmicutes__g__Murimonas__s__intestini_00000003	0,14	1	0,96	25,62	36,58	26,89	41,64	2,151303263	3,191929722	-1,428
p__Firmicutes__g__Clostridium__s__sp._00000100	0,14	1	0,96	0	4,53	0	0	0	0	-18500
p__Firmicutes__g__Anaerovorax__s__odorimutans_00000332	0,14	1	0,96	0	2,09	0	0	0	0	-8538
p__Actinobacteria__g__Adlercreutzia__s__equolifaciens_00000134	0,14	1	0,96	11,82	9,48	11,7	9	0,93604493	0,689898355	1,247
p__Bacteroidetes__o__Bacteroidales_00000176	0,15	1	0,96	2,67	5,73	0	7,29	0	0,558817668	-2,15
p__Firmicutes__o__Clostridiales_00000259	0,16	1	0,96	1,11	3,2	0	3,8	0	0,291290417	-2,895
p__Firmicutes__o__Clostridiales_00000068	0,17	1	0,96	5,46	8,61	5,2	7,68	0,416019969	0,588713263	-1,578
p__Firmicutes__g__Eubacterium__s__oxidoreducens_00000116	0,17	1	0,96	6,04	5,5	5,48	4,95	0,438421044	0,379444095	1,098
p__Firmicutes__o__Clostridiales_00000015	0,18	1	0,96	14,2	18,47	12,85	18,18	1,028049346	1,393594677	-1,301
p__Firmicutes__o__Clostridiales_00000005	0,18	1	0,96	18,56	24,59	17,64	25,24	1,411267741	1,934781609	-1,325
p__Firmicutes__g__Ruminiclostridium__s__Eubacterium_siraeum_00000138	0,18	1	0,96	3,36	9,27	0	9,26	0	0,709828752	-2,762
p__Firmicutes__g__Lachnoclostridium__s__Clostridium_saccharolyticum_00000023	0,18	1	0,96	12,36	18,77	13,45	22,62	1,07605165	1,733944532	-1,518
p__Firmicutes__g__Eubacterium__s__desmolans_00000156	0,19	1	0,96	1,27	4,33	0	3,16	0	0,242230978	-3,409
p__Firmicutes__g__Dehalobacterium__s__formicoaceticum_00000088	0,2	1	0,96	3,57	6,66	3,61	5,14	0,288813863	0,394008616	-1,864
p__Firmicutes__g__Clostridium__s__sp._BSY12_00000139	0,2	1	0,96	4,42	1,78	5	0	0,400019201	0	2,485
p__Verrucomicrobia__g__Akkermansia_00000428	0,21	1	0,96	1,56	0	0	0	0	0	6357
p___Unclassified.Bacteria__c___00000256	0,21	1	0,96	2,94	0	0	0	0	0	11990
p__Firmicutes__g__Ruminococcus__s__sp._00000362	0,21	1	0,96	2,35	0	0	0	0	0	9604
p__Firmicutes__g__Ruminococcus__s__bicirculans_00000376	0,21	1	0,96	2,26	0	0	0	0	0	9225
p__Firmicutes__g__Ruminiclostridium__s__Eubacterium_siraeum_00000199	0,21	1	0,96	4,49	0	0	0	0	0	18350
p__Firmicutes__g__Roseburia_00000381	0,21	1	0,96	2,17	0	0	0	0	0	8846
p__Firmicutes__g__Oscillibacter__s__sp._G2_00000468	0,21	1	0,96	1,31	0	0	0	0	0	5342
p__Firmicutes__g__Holdemanella__s__biformis_00000311	0,21	1	0,96	1,48	0	0	0	0	0	6059
p__Firmicutes__g__Faecalibaculum__s__rodentium_00000383	0,21	1	0,96	1,27	0	0	0	0	0	5202
p__Firmicutes__g__Faecalibaculum__s__rodentium_00000377	0,21	1	0,96	1,28	0	0	0	0	0	5231
p__Firmicutes__g__Eubacterium__s__ramulus_00000416	0,21	1	0,96	1,38	0	0	0	0	0	5645
p__Firmicutes__g__Eubacterium__s__ramulus_00000231	0,21	1	0,96	2,39	0	0	0	0	0	9750
p__Firmicutes__g__Dorea__s__formicigenerans_00000361	0,21	1	0,96	3,03	0	0	0	0	0	12360
p__Firmicutes__g__Clostridium__s__sp._Culture1_00000387	0,21	1	0,96	1,21	0	0	0	0	0	4940
p__Firmicutes__g__Clostridium__s__sp._Clone27_00000391	0,21	1	0,96	1,21	0	0	0	0	0	4946
p__Firmicutes__g__Clostridium__s__sp._Clone27_00000341	0,21	1	0,96	1,29	0	0	0	0	0	5249
p__Firmicutes__g__Clostridium__s__sp._AT5_00000304	0,21	1	0,96	1,51	0	0	0	0	0	6153
p__Firmicutes__g__Clostridium_00000336	0,21	1	0,96	1,39	0	0	0	0	0	5692
p__Firmicutes__f__Ruminococcaceae_00000363	0,21	1	0,96	2,48	0	0	0	0	0	10140
p__Bacteroidetes__o__Bacteroidales_00000150	0,22	1	0,96	4,82	6,18	6,08	8,27	0,486423348	0,633939933	-1,283
p__Bacteroidetes__g__Alistipes__s__massiliensis_00000026	0,22	1	0,96	13,66	21,03	12,81	20,92	1,024849193	1,603630398	-1,539
p__Firmicutes__o__Clostridiales_00000149	0,23	1	0,96	5,42	2,27	6,48	0	0,518424884	0	2,385
p__Firmicutes__o__Clostridiales_00000013	0,23	1	0,96	14,45	20,38	14,7	19,84	1,176056451	1,520842596	-1,411
p__Firmicutes__g__Clostridium__s__sp._Culture54_00000056	0,23	1	0,96	7,57	5,38	7,07	4,64	0,56562715	0,35568093	1,407
p__Firmicutes__g__Clostridium__s__sp._AT5_00000059	0,23	1	0,96	5,66	7,26	5,48	7,42	0,438421044	0,568782866	-1,284
p__Actinobacteria__g__Olsenella_00000030	0,23	1	0,96	23,32	17,59	23,81	16,45	1,904891435	1,260980882	1,326
p__Firmicutes__g__Lachnoclostridium__s__Clostridium_saccharolyticum_00000107	0,24	1	0,96	3,87	6,62	0	6,88	0	0,527388965	-1,711
p__Firmicutes__g__Eubacterium__s__sp._MarseilleP3254_00000241	0,25	1	0,96	1,28	2,75	0	2,35	0	0,180140126	-2,152
p__Firmicutes__g__Ruthenibacterium__s__lactatiformans_00000102	0,26	1	0,96	6,9	5,58	6,93	0	0,554426612	0	1,236
p__Firmicutes__g__Ruminiclostridium__s__thermocellum_00000237	0,26	1	0,96	4,27	1,1	0	0	0	0	3,866
p__Firmicutes__g__Eubacterium__s__desmolans_00000152	0,26	1	0,96	1,38	4,41	0	3,2	0	0,245297193	-3,202
p__Firmicutes__g__Clostridium__s__sp._BSY12_00000228	0,26	1	0,96	2,24	0,6	0	0	0	0	3,717
k__Unclassified_00000234	0,26	1	0,96	3,69	1,6	3,32	0	0,265612749	0	2,313
p__Bacteroidetes__g__Prevotella__s__dentalis_00000092	0,27	1	0,96	7,67	4,37	6,63	2,12	0,53042546	0,16250939	1,756
p__Firmicutes__g__Clostridium_00000161	0,29	1	0,96	1,82	3,87	0	4,13	0	0,31658669	-2,128
p__Firmicutes__o__Clostridiales_00000239	0,31	1	0,96	1,5	3,2	0	3,74	0	0,286691094	-2,134
p__Firmicutes__g__Moryella__s__indoligenes_00000081	0,31	1	0,96	5,87	9,12	5,2	9,82	0,416019969	0,752755761	-1,554
p__Deferribacteres__g__Mucispirillum__s__schaedleri_00000143	0,31	1	0,96	4,33	6,06	0	7,4	0	0,567249759	-1,401
p__Firmicutes__g__Clostridium__s__sp._40ccB5824ARE_00000054	0,32	1	0,96	6,28	7,61	5,48	7,88	0,438421044	0,604044337	-1,211
p__Firmicutes__f__Lachnospiraceae_00000032	0,32	1	0,96	13,52	13,93	10,1	14,97	0,808038786	1,14753093	-1,03
p__Firmicutes__g__Stomatobaculum__s__longum_00000158	0,33	1	0,96	5,63	2,25	8,83	0	0,706433909	0	2,496
p__Firmicutes__g__Clostridium__s__sp._AT5_00000154	0,33	1	0,96	3,05	1,72	4,24	0	0,339216282	0	1,779
p__Streptophyta__f__Fabaceae_00000335	0,34	1	0,96	3,92	1,49	0	0	0	0	2,633
p__Firmicutes__o__Clostridiales_00000206	0,34	1	0,96	1,39	2,86	0	2	0	0,153310746	-2,06
p__Firmicutes__g__Clostridium__s__sp._BSY12_00000284	0,34	1	0,96	1,82	0,5	0	0	0	0	3,636
p__Firmicutes__g__Clostridium__s__sp._BSY12_00000208	0,34	1	0,96	2,45	0,69	0	0	0	0	3,569
p__Verrucomicrobia__g__Akkermansia_00000418	0,35	1	0,96	0	1,31	0	0	0	0	-5355
p__Spirochaetes__g__Treponema__s__succinifaciens_00000642	0,35	1	0,96	0	1,2	0	0	0	0	-4906
p__Nematoda__g__Haemonchus__s__placei_00000349	0,35	1	0,96	0	2,96	0	0	0	0	-12080
p__Firmicutes__o__Clostridiales_00000456	0,35	1	0,96	0	1,1	0	0	0	0	-4511
p__Firmicutes__o__Clostridiales_00000443	0,35	1	0,96	0	1,13	0	0	0	0	-4613
p__Firmicutes__o__Clostridiales_00000426	0,35	1	0,96	0	1,18	0	0	0	0	-4811
p__Firmicutes__o__Clostridiales_00000403	0,35	1	0,96	0	1,25	0	0	0	0	-5089
p__Firmicutes__o__Clostridiales_00000250	0,35	1	0,96	0	1,96	0	0	0	0	-8021
p__Firmicutes__o__Clostridiales_00000227	0,35	1	0,96	0	2,17	0	0	0	0	-8845
p__Firmicutes__g__Ruminococcus__s__sp._MarseilleP3217_00000528	0,35	1	0,96	0	1,33	0	0	0	0	-5443
p__Firmicutes__g__Ruminococcus__s__sp._16442_00000669	0,35	1	0,96	0	1	0	0	0	0	-4083
p__Firmicutes__g__Ruminococcus__s__sp._00000497	0,35	1	0,96	0	1,42	0	0	0	0	-5811
p__Firmicutes__g__Ruminococcus__s__sp._00000494	0,35	1	0,96	0	1,43	0	0	0	0	-5851
p__Firmicutes__g__Ruminococcus__s__sp._00000472	0,35	1	0,96	0	1,52	0	0	0	0	-6198
p__Firmicutes__g__Ruminococcus__s__sp._00000434	0,35	1	0,96	0	1,66	0	0	0	0	-6770
p__Firmicutes__g__Ruminococcus_00000414	0,35	1	0,96	0	1,72	0	0	0	0	-7035
p__Firmicutes__g__Ruminococcus_00000320	0,35	1	0,96	0	2,21	0	0	0	0	-9028
p__Firmicutes__g__Ruminococcus_00000303	0,35	1	0,96	0	2,3	0	0	0	0	-9402
p__Firmicutes__g__Ruminiclostridium__s__sp._KB18_00000589	0,35	1	0,96	0	1,25	0	0	0	0	-5089
p__Firmicutes__g__Oscillibacter__s__sp._MarseilleP2778_00000354	0,35	1	0,96	0	1,39	0	0	0	0	-5695
p__Firmicutes__g__Murimonas__s__intestini_00000625	0,35	1	0,96	0	1	0	0	0	0	-4083
p__Firmicutes__g__Murimonas__s__intestini_00000600	0,35	1	0,96	0	1,04	0	0	0	0	-4253
p__Firmicutes__g__Murimonas__s__intestini_00000595	0,35	1	0,96	0	1,04	0	0	0	0	-4253
p__Firmicutes__g__Murimonas__s__intestini_00000587	0,35	1	0,96	0	1,05	0	0	0	0	-4300
p__Firmicutes__g__Murimonas__s__intestini_00000567	0,35	1	0,96	0	1,09	0	0	0	0	-4464
p__Firmicutes__g__Murimonas__s__intestini_00000555	0,35	1	0,96	0	1,12	0	0	0	0	-4566
p__Firmicutes__g__Murimonas__s__intestini_00000554	0,35	1	0,96	0	1,13	0	0	0	0	-4613
p__Firmicutes__g__Murimonas__s__intestini_00000533	0,35	1	0,96	0	1,19	0	0	0	0	-4858
p__Firmicutes__g__Murimonas__s__intestini_00000489	0,35	1	0,96	0	1,31	0	0	0	0	-5355
p__Firmicutes__g__Lachnoclostridium__s__Clostridium_saccharolyticum_00000248	0,35	1	0,96	0	2,54	0	0	0	0	-10360

p__Firmicutes__g__Lachnoclostridium__s__Clostridium_asparagiforme_00000620	0,35	1	0,96	0	1	0	0	0	0	-4083
p__Firmicutes__g__Lachnoclostridium__s__Clostridium_asparagiforme_00000585	0,35	1	0,96	0	1,05	0	0	0	0	-4300
p__Firmicutes__g__Lachnoclostridium__s__Clostridium_asparagiforme_00000430	0,35	1	0,96	0	1,49	0	0	0	0	-6083
p__Firmicutes__g__Lachnoclostridium__s__Clostridium_asparagiforme_00000415	0,35	1	0,96	0	1,54	0	0	0	0	-6307
p__Firmicutes__g__Lachnoclostridium__s__Clostridium_asparagiforme_00000397	0,35	1	0,96	0	1,62	0	0	0	0	-6600
p__Firmicutes__g__Lachnoclostridium_00000624	0,35	1	0,96	0	1	0	0	0	0	-4083
p__Firmicutes__g__Lachnoclostridium_00000601	0,35	1	0,96	0	1,03	0	0	0	0	-4191
p__Firmicutes__g__Lachnoclostridium_00000560	0,35	1	0,96	0	1,12	0	0	0	0	-4566
p__Firmicutes__g__Lachnoclostridium_00000532	0,35	1	0,96	0	1,19	0	0	0	0	-4858
p__Firmicutes__g__Lachnoanaerobaculum__s__umeaense_00000581	0,35	1	0,96	0	1,07	0	0	0	0	-4355
p__Firmicutes__g__Lachnoanaerobaculum__s__umeaense_00000455	0,35	1	0,96	0	1,4	0	0	0	0	-5736
p__Firmicutes__g__Lachnoanaerobaculum__s__umeaense_00000427	0,35	1	0,96	0	1,5	0	0	0	0	-6123
p__Firmicutes__g__Eubacterium__s__ramulus_00000450	0,35	1	0,96	0	1,04	0	0	0	0	-4253
p__Firmicutes__g__Eubacterium__s__plexicaudatum_00000181	0,35	1	0,96	0	2,56	0	0	0	0	-10450
p__Firmicutes__g__Desulfotomaculum__s__sp._SN1_H_00000192	0,35	1	0,96	0	2,09	0	0	0	0	-8525
p__Firmicutes__g__Coprococcus__s__catus_00000517	0,35	1	0,96	0	1,96	0	0	0	0	-7994
p__Firmicutes__g__Coprococcus__s__catus_00000299	0,35	1	0,96	0	3,38	0	0	0	0	-13780
p__Firmicutes__g__Clostridium__s__sp._Clone27_00000115	0,35	1	0,96	0	2,99	0	0	0	0	-12210
p__Firmicutes__g__Clostridium__s__sp._00000230	0,35	1	0,96	0	1,63	0	0	0	0	-6668
p__Firmicutes__g__Clostridium_00000167	0,35	1	0,96	0	2,33	0	0	0	0	-9498
p__Firmicutes__f__Lachnospiraceae_00000356	0,35	1	0,96	0	1,78	0	0	0	0	-7266
p__Firmicutes__f__Clostridiaceae_00000466	0,35	1	0,96	0	1	0	0	0	0	-4083
p__Firmicutes__f__Clostridiaceae_00000454	0,35	1	0,96	0	1,03	0	0	0	0	-4191
p__Firmicutes__f__Clostridiaceae_00000448	0,35	1	0,96	0	1,03	0	0	0	0	-4191
p__Firmicutes__f__Clostridiaceae_00000411	0,35	1	0,96	0	1,12	0	0	0	0	-4566
p__Firmicutes__f__Clostridiaceae_00000350	0,35	1	0,96	0	1,3	0	0	0	0	-5314
p__Firmicutes__f__Clostridiaceae_00000340	0,35	1	0,96	0	1,33	0	0	0	0	-5443
p__Firmicutes__f__Clostridiaceae_00000325	0,35	1	0,96	0	1,42	0	0	0	0	-5777
p__Firmicutes__f__Clostridiaceae_00000308	0,35	1	0,96	0	1,48	0	0	0	0	-6049
p__Firmicutes__f__Clostridiaceae_00000290	0,35	1	0,96	0	1,54	0	0	0	0	-6307
p__Firmicutes__f__Clostridiaceae_00000281	0,35	1	0,96	0	1,61	0	0	0	0	-6559
p__Firmicutes__f__Clostridiaceae_00000028	0,35	1	0,96	0	6,89	0	0	0	0	-28120
p__Firmicutes__c___00000396	0,35	1	0,96	0	1,01	0	0	0	0	-4137
p__Actinobacteria__g__Asaccharobacter__s__sp._WCA131CoC2_00000050	0,35	1	0,96	16,69	18,9	15,13	19,67	1,210458102	1,507811182	-1,133
p__Firmicutes__o__Lactobacillales_00000002	0,37	1	0,96	28,52	24,11	24,66	20,82	1,972894699	1,595964861	1,183
p__Firmicutes__g__Eubacterium_00000062	0,37	1	0,96	8,53	6,52	7,07	6,24	0,56562715	0,478329526	1,308
p__Firmicutes__c___00000042	0,37	1	0,96	7,85	8,82	7,94	8,54	0,635230491	0,654636883	-1,124
p__Bacteroidetes__g__Alistipes_00000018	0,37	1	0,96	19,47	24,5	19,7	29,58	1,576075652	2,267465927	-1,258
p__Actinobacteria__g__Eggerthella__s__sp._YY7918_00000076	0,37	1	0,96	12,7	15,81	14,46	15,7	1,156855529	1,203489353	-1,245
p__Verrucomicrobia__g__Akkermansia_00000469	0,38	1	0,96	0,49	1,45	0	0	0	0	-2,939
p__Verrucomicrobia__g__Akkermansia_00000352	0,38	1	0,96	0,62	1,88	0	0	0	0	-3,01
p__Firmicutes__g__Intestinimonas_00000232	0,38	1	0,96	0,64	2,65	0	0	0	0	-4,143
p__Firmicutes__g__Clostridium__s__sp._Culture41_00000279	0,38	1	0,96	0,47	1,78	0	0	0	0	-3,745
p__Firmicutes__g__Clostridium__s__sp._BNL1100_00000297	0,38	1	0,96	0,49	1,6	0	0	0	0	-3,226
k__Unclassified_00000457	0,38	1	0,96	0,43	1,53	0	0	0	0	-3,561
p__Firmicutes__o__Clostridiales_00000120	0,39	1	0,96	6,25	4,49	6,56	5,18	0,524825192	0,397074831	1,394
p__Firmicutes__g__Eisenbergiella__s__sp._AT9_00000106	0,39	1	0,96	8,28	5,76	8,19	6,94	0,655231451	0,531988287	1,436
p__Firmicutes__g__Christensenella__s__sp._MarseilleP2438_00000285	0,39	1	0,96	2,45	1,41	3,74	0	0,299214362	0	1,734
p__Actinobacteria__g__Enterorhabdus__s__caecimuris_00000137	0,39	1	0,96	10,88	7,82	8,54	7,52	0,683232795	0,576448403	1,391
p__Firmicutes__g__Clostridium__s__sp._Clone27_00000095	0,41	1	0,96	4,84	2,55	4,47	0	0,357617166	0	1,896
p__Firmicutes__o__Lactobacillales_00000027	0,43	1	0,96	13,44	11,47	11,27	9,73	0,901643279	0,745856777	1,171
p__Firmicutes__g__Ruminococcus__s__sp._MarseilleP3217_00000061	0,43	1	0,96	9,31	13,59	11,36	12,61	0,908843624	0,966624251	-1,459
p__Firmicutes__g__Oscillibacter__s__sp._MarseilleP2778_00000224	0,43	1	0,96	2,78	0,93	0	0	0	0	2,999
p__Firmicutes__g__Johnsonella__s__ignava_00000246	0,43	1	0,96	5,38	2,1	0	0	0	0	2,56
p__Firmicutes__g__Clostridium__s__sp._Clone27_00000223	0,43	1	0,96	2,14	0,9	0	0	0	0	2,383
p__Firmicutes__g__Clostridium__s__sp._A9_00000044	0,43	1	0,96	5,65	7,19	4,36	6,28	0,348816743	0,481395741	-1,273
p__Firmicutes__c___00000291	0,43	1	0,96	1,69	0,71	0	0	0	0	2,385
k__Unclassified_00000128	0,43	1	0,96	6,48	4,56	6,32	4,95	0,50562427	0,379444095	1,42
p__Proteobacteria__g__Parasutterella__s__excrementihominis_00000558	0,44	1	0,96	0,87	0	0	0	0	0	3546
p__Proteobacteria__g__Parasutterella__s__excrementihominis_00000515	0,44	1	0,96	0,95	0	0	0	0	0	3867
p__Firmicutes__o__Lactobacillales_00000534	0,44	1	0,96	0,9	0	0	0	0	0	3686
p__Firmicutes__o__Lactobacillales_00000518	0,44	1	0,96	0,94	0	0	0	0	0	3826
p__Firmicutes__o__Lactobacillales_00000488	0,44	1	0,96	1,01	0	0	0	0	0	4123
p__Firmicutes__o__Lactobacillales_00000486	0,44	1	0,96	1,01	0	0	0	0	0	4123
p__Firmicutes__o__Lactobacillales_00000461	0,44	1	0,96	1,07	0	0	0	0	0	4363
p__Firmicutes__o__Clostridiales_00000380	0,44	1	0,96	1,12	0	0	0	0	0	4555
p__Firmicutes__o__Clostridiales_00000324	0,44	1	0,96	1,32	0	0	0	0	0	5377
p__Firmicutes__o__Clostridiales_00000245	0,44	1	0,96	1,75	0	0	0	0	0	7144
p__Firmicutes__o__Clostridiales_00000144	0,44	1	0,96	2,9	0	0	0	0	0	11820
p__Firmicutes__o__Clostridiales_00000110	0,44	1	0,96	3,38	0	0	0	0	0	13800
p__Firmicutes__g__Turicibacter_00000406	0,44	1	0,96	0,86	0	0	0	0	0	3500
p__Firmicutes__g__Ruthenibacterium__s__lactatiformans_00000478	0,44	1	0,96	1,36	0	0	0	0	0	5564
p__Firmicutes__g__Ruminococcus__s__bicirculans_00000441	0,44	1	0,96	1,4	0	0	0	0	0	5715
p__Firmicutes__g__Ruminococcus__s__bicirculans_00000373	0,44	1	0,96	1,62	0	0	0	0	0	6625
p__Firmicutes__g__Ruminococcus__s__bicirculans_00000301	0,44	1	0,96	1,98	0	0	0	0	0	8083
p__Firmicutes__g__Ruminiclostridium__s__Clostridium_cellulosi_00000654	0,44	1	0,96	0,94	0	0	0	0	0	3826
p__Firmicutes__g__Ruminiclostridium__s__Clostridium_cellulosi_00000611	0,44	1	0,96	1,02	0	0	0	0	0	4164
p__Firmicutes__g__Ruminiclostridium__s__Clostridium_cellulolyticum_00000471	0,44	1	0,96	1,39	0	0	0	0	0	5657
p__Firmicutes__g__Ruminiclostridium__s__Clostridium_cellobioparum_00000524	0,44	1	0,96	1,21	0	0	0	0	0	4951
p__Firmicutes__g__Roseburia_00000487	0,44	1	0,96	1,21	0	0	0	0	0	4951
p__Firmicutes__g__Roseburia_00000481	0,44	1	0,96	1,24	0	0	0	0	0	5051
p__Firmicutes__g__Roseburia_00000368	0,44	1	0,96	1,57	0	0	0	0	0	6415
p__Firmicutes__g__Oxobacter__s__sp._PPf50E4_00000465	0,44	1	0,96	0,86	0	0	0	0	0	3500
p__Firmicutes__g__Oxobacter__s__sp._PPf50E4_00000462	0,44	1	0,96	0,86	0	0	0	0	0	3500
p__Firmicutes__g__Oxobacter__s__sp._PPf50E4_00000328	0,44	1	0,96	1,2	0	0	0	0	0	4916
p__Firmicutes__g__Lactobacillus_00000353	0,44	1	0,96	1,06	0	0	0	0	0	4328
p__Firmicutes__g__Johnsonella__s__sp._oral_taxon_166_00000605	0,44	1	0,96	1,43	0	0	0	0	0	5832
p__Firmicutes__g__Holdemanella__s__biformis_00000084	0,44	1	0,96	3,14	0	0	0	0	0	12800
p__Firmicutes__g__Clostridium__s__sp._AT5_00000202	0,44	1	0,96	1,63	0	0	0	0	0	6648
p__Firmicutes__g__Clostridium__s__sp._AT5_00000060	0,44	1	0,96	3,62	0	0	0	0	0	14760
p__Firmicutes__g__Clostridium_00000334	0,44	1	0,96	0,99	0	0	0	0	0	4042
p__Firmicutes__g__Clostridium_00000117	0,44	1	0,96	2,55	0	0	0	0	0	10410
p__Firmicutes__g__Blautia__s__Ruminococcus_gnavus_00000596	0,44	1	0,96	1,01	0	0	0	0	0	4123
p__Firmicutes__f__Lachnospiraceae_00000423	0,44	1	0,96	1,29	0	0	0	0	0	5284
p__Firmicutes__f__Lachnospiraceae_00000375	0,44	1	0,96	1,44	0	0	0	0	0	5890
p__Firmicutes__c___00000189	0,44	1	0,96	1,78	0	0	0	0	0	7260
p__Bacteroidetes__g__Prevotella__s__denticola_00000604	0,44	1	0,96	0,87	0	0	0	0	0	3546
p__Bacteroidetes__g__Alistipes__s__massiliensis_00000592	0,44	1	0,96	1,14	0	0	0	0	0	4666
p__Bacteroidetes__g__Alistipes__s__massiliensis_00000504	0,44	1	0,96	1,37	0	0	0	0	0	5593
p__Bacteroidetes__g__Alistipes__s__massiliensis_00000355	0,44	1	0,96	1,94	0	0	0	0	0	7931
p__Actinobacteria__g__Bifidobacterium__s__pseudolongum_00000530	0,44	1	0,96	1,02	0	0	0	0	0	4164
p__Actinobacteria__g__Bifidobacterium__s__pseudolongum_00000453	0,44	1	0,96	1,2	0	0	0	0	0	4916
p__Actinobacteria__g__Bifidobacterium__s__pseudolongum_00000382	0,44	1	0,96	1,41	0	0	0	0	0	5774
p__Actinobacteria__g__Bifidobacterium__s__pseudolongum_00000378	0,44	1	0,96	1,43	0	0	0	0	0	5832
p__Actinobacteria__g__Bifidobacterium__s__pseudolongum_00000347	0,44	1	0,96	1,55	0	0	0	0	0	6333
p__Actinobacteria__g__Bifidobacterium__s__pseudolongum_00000329	0,44	1	0,96	1,65	0	0	0	0	0	6724
p__Firmicutes__g__Ruminiclostridium__s__Clostridium_leptum_00000113	0,45	1	0,98	8,74	10,42	8,66	9,89	0,692833256	0,758121637	-1,193
p__Firmicutes__g__Moryella__s__indoligenes_00000022	0,45	1	0,98	14,44	17,43	14,32	17,66	1,145654991	1,353733883	-1,207
p__Proteobacteria__g__Parasutterella__s__excrementihominis_00000122	0,47	1	1	6,99	4,3	6,25	5,82	0,500024001	0,44613427	1,625
p__Firmicutes__g__Lachnoclostridium__s__Clostridium_saccharolyticum_00000064	0,47	1	1	10,21	10,67	8,77	9,13	0,701633678	0,699863553	-1,045
p__Firmicutes__g__Coprococcus__s__catus_00000124	0,47	1	1	9,14	13,88	11,14	12,94	0,89124278	0,991920524	-1,518
p__Firmicutes__o__Clostridiales_00000183	0,48	1	1	3,9	1,76	4,58	0	0,366417588	0	2,212
p__Firmicutes__g__Pseudoflavonifractor__s__sp._MarseilleP3106_00000196	0,48	1	1	3,3	2,12	4,58	0	0,366417588	0	1,557
p__Firmicutes__g__Clostridium__s__sp._Clone27_00000155	0,48	1	1	3,42	1,92	4,12	0	0,329615822	0	1,776
p__Firmicutes__g__Blautia__s__Ruminococcus_gnavus_00000261	0,48	1	1	3,6	2,31	5,57	0	0,44562139	0	1,558
p__Verrucomicrobia__g__Akkermansia_00000431	0,5	1	1	0,64	1,46	0	0	0	0	-2,288
p__Verrucomicrobia__g__Akkermansia_00000358	0,5	1	1	0,77	1,7	0	0	0	0	-2,21
p__Verrucomicrobia__g__Akkermansia_00000344	0,5	1	1	0,82	1,74	0	0	0	0	-2,116
p__Verrucomicrobia__g__Akkermansia_00000331	0,5	1	1	0,83	1,86	0	0	0	0	-2,237
p__Verrucomicrobia__g__Akkermansia_00000242	0,5	1	1	0,9	2,69	0	0	0	0	-2,983
p__Firmicutes__o__Clostridiales_00000317	0,5	1	1	0,85	1,72	0	0	0	0	-2,033
p__Firmicutes__g__Clostridium__s__sp._A9_00000118	0,5	1	1	1,6	3,14	0	0	0	0	-1,966

p__Firmicutes__g__Clostridium_00000263	0,5	1	1	0,4	1,73	0	0	0	0	-4,285
p__Firmicutes__g__Anaerotaenia__s__torta_00000173	0,5	1	1	1,82	4,06	0	0	0	0	-2,233
p__Firmicutes__f__Ruminococcaceae_00000157	0,51	1	1	5,14	6,98	7,75	7,87	0,620029761	0,603277784	-1,357
p__Firmicutes__g__Clostridium_00000080	0,52	1	1	4,5	6,24	5,39	6,15	0,431220699	0,471430543	-1,387
p__Firmicutes__g__Oscillospira__s__guilliermondii_00000047	0,53	1	1	9,29	13,44	11,75	12,07	0,940045122	0,925230349	-1,447
p__Firmicutes__g__Clostridium__s__sp._Culture41_00000012	0,53	1	1	13,37	14,88	11,18	13,45	0,894442933	1,031014764	-1,113
p__Firmicutes__g__Clostridium__s__sp._BSY12_00000004	0,53	1	1	19,09	14,73	22,07	13,91	1,765684753	1,066276235	1,296
p__Actinobacteria__g__Asaccharobacter__s__sp._WCA131CoC2_00000069	0,53	1	1	14,79	16,11	13,82	15,13	1,105653071	1,15979579	-1,089
p__Firmicutes__g__Ruminococcus__s__sp._MarseilleP3217_00000264	0,54	1	1	3,15	1,51	0	0	0	0	2,085
p__Firmicutes__g__Robinsoniella__s__peoriensis_00000252	0,54	1	1	2,71	1,7	0	0	0	0	1,597
p__Firmicutes__o__Clostridiales_00000078	0,56	1	1	5,29	1,75	0	0	0	0	3,028
p__Firmicutes__g__Ruminiclostridium__s__thermocellum_00000193	0,56	1	1	4,01	1,8	0	0	0	0	2,235
p__Firmicutes__g__Intestinimonas_00000184	0,56	1	1	4,08	2,76	4,47	2,55	0,357617166	0,195471201	1,476
p__Firmicutes__g__Faecalibaculum__s__rodentium_00000310	0,56	1	1	1,35	0,62	0	0	0	0	2,163
p__Firmicutes__g__Faecalibaculum__s__rodentium_00000288	0,56	1	1	1,49	0,58	0	0	0	0	2,591
p__Firmicutes__g__Faecalibaculum__s__rodentium_00000274	0,56	1	1	1,48	0,76	0	0	0	0	1,94
p__Firmicutes__g__Clostridium__s__sp._MarseilleP2776_00000142	0,56	1	1	3,94	3,26	4,9	4,36	0,392018817	0,334217425	1,208
p__Firmicutes__g__Clostridium__s__sp._Culture57_00000104	0,56	1	1	3,3	1,26	0	0	0	0	2,622
p__Firmicutes__g__Clostridium__s__sp._Culture41_00000126	0,56	1	1	3,01	4,57	3,46	4,06	0,276813287	0,311220813	-1,518
p__Firmicutes__g__Clostridium_00000326	0,56	1	1	0,64	1,3	0	0	0	0	-2,033
p__Firmicutes__c___00000330	0,56	1	1	0,65	1,34	0	0	0	0	-2,048
p__Actinobacteria__g__Bifidobacterium__s__pseudolongum_00000037	0,56	1	1	9,63	3,17	0	0	0	0	3,04
p__Verrucomicrobia__g__Akkermansia_00000240	0,57	1	1	1,82	2,85	0	2,64	0	0,202370184	-1,563
p__Firmicutes__o__Clostridiales_00000066	0,57	1	1	7,03	8,63	7,07	8,61	0,56562715	0,66000276	-1,229
p__Firmicutes__o__Clostridiales_00000051	0,57	1	1	6,82	8,73	6,4	7,23	0,512024577	0,554218345	-1,281
p__Firmicutes__g__Eubacterium__s__sp._15_00000194	0,59	1	1	2,15	3,16	0	2,79	0	0,21386849	-1,469
p__Firmicutes__g__Clostridium__s__sp._Clone3_00000175	0,59	1	1	2,85	1,8	3,32	0	0,265612749	0	1,584
p__Firmicutes__g__Clostridium__s__sp._A9_00000197	0,59	1	1	1,76	2,54	0	3,38	0	0,25909516	-1,442
p__Firmicutes__g__Clostridium__s__sp._PPf35E6_00000203	0,6	1	1	1,8	2,79	0	3,24	0	0,248363408	-1,553
p__Firmicutes__g__Clostridium__s__sp._Culture54_00000165	0,6	1	1	2,29	3,28	0	3,23	0	0,247596854	-1,43
p__Firmicutes__o__Clostridiales_00000074	0,61	1	1	6,82	5,12	8,12	3,04	0,649631182	0,233032333	1,333
p__Firmicutes__g__Clostridium__s__sp._SN17_00000160	0,61	1	1	3,33	2,53	3,87	3,38	0,309614862	0,25909516	1,314
p__Firmicutes__g__Anaerotaenia__s__torta_00000148	0,61	1	1	6,03	4,43	7	4,95	0,560026881	0,379444095	1,36
p__Firmicutes__g__Anaerovorax__s__odorimutans_00000098	0,62	1	1	7,08	6,17	7,14	6,96	0,571227419	0,533521395	1,148
p__Verrucomicrobia__g__Akkermansia_00000470	0,63	1	1	0,61	1,31	0	0	0	0	-2,162
p__Verrucomicrobia__g__Akkermansia_00000442	0,63	1	1	1,26	0,71	0	0	0	0	1,783
p__Verrucomicrobia__g__Akkermansia_00000419	0,63	1	1	0,7	1,45	0	0	0	0	-2,076
p__Verrucomicrobia__g__Akkermansia_00000401	0,63	1	1	0,73	1,51	0	0	0	0	-2,07
p__Verrucomicrobia__g__Akkermansia_00000374	0,63	1	1	0,76	1,63	0	0	0	0	-2,157
p__Verrucomicrobia__g__Akkermansia_00000370	0,63	1	1	0,78	1,59	0	0	0	0	-2,029
p__Verrucomicrobia__g__Akkermansia_00000364	0,63	1	1	0,78	1,65	0	0	0	0	-2,105
p__Verrucomicrobia__g__Akkermansia_00000287	0,63	1	1	1,08	1,93	0	0	0	0	-1,792
p__Proteobacteria__o__Desulfovibrionales_00000055	0,63	1	1	11,39	14,14	11	15,45	0,880042242	1,184325509	-1,242
p__Firmicutes__g__Turicibacter_00000021	0,63	1	1	4,32	4,91	0	0	0	0	-1,138
p__Firmicutes__g__Clostridium__s__sp._SN17_00000348	0,63	1	1	0,67	1,12	0	0	0	0	-1,676
p__Firmicutes__g__Clostridium__s__sp._ASF502_00000009	0,63	1	1	15,77	11,88	13,42	11,71	1,073651535	0,897634415	1,327
p__Firmicutes__g__Ruminiclostridium__s__Clostridium_cellulolyticum_00000198	0,65	1	1	4,5	4,43	7,21	3,5	0,576827688	0,268293805	1,016
p__Firmicutes__g__Lactobacillus_00000079	0,65	1	1	5,31	4,23	6,08	2,12	0,486423348	0,16250939	1,256
p__Verrucomicrobia__g__Akkermansia_00000338	0,66	1	1	1,14	1,81	0	0	0	0	-1,587
p__Verrucomicrobia__g__Akkermansia_00000251	0,66	1	1	1,81	2,19	0	0	0	0	-1,211
p__Verrucomicrobia__g__Akkermansia_00000164	0,66	1	1	2,64	3,5	0	0	0	0	-1,328
p__Firmicutes__o__Clostridiales_00000052	0,66	1	1	6,37	8,13	6,16	5,33	0,492823656	0,408573137	-1,275
p__Firmicutes__g__Anaerotaenia__s__torta_00000067	0,66	1	1	9,17	8,3	11,49	8,73	0,919244124	0,669201404	1,105
p__Firmicutes__g__Clostridium__s__sp._Culture41_00000090	0,67	1	1	5,47	4,78	4,9	5,11	0,392018817	0,391708955	1,145
p__Firmicutes__g__Clostridium__s__sp._Culture41_00000040	0,67	1	1	7,4	8,78	7,28	8,04	0,582427957	0,616309197	-1,187
p__Bacteroidetes__g__Parabacteroides__s__sp._YL27_00000221	0,67	1	1	2,84	2,61	3,32	2,74	0,265612749	0,210035721	1,088
p__Actinobacteria__g__Enterorhabdus__s__mucosicola_00000065	0,67	1	1	15,31	16,54	14,49	16,52	1,159255644	1,266346758	-1,08
p__Firmicutes__o__Clostridiales_00000218	0,68	1	1	2,59	1,94	0	0	0	0	1,333
p__Firmicutes__g__Clostridium_00000257	0,68	1	1	1,72	1,25	0	0	0	0	1,383
p__Firmicutes__g__Clostridium_00000219	0,68	1	1	2,09	1,34	0	0	0	0	1,563
p__Firmicutes__c___00000269	0,68	1	1	1,83	1,1	0	0	0	0	1,662
p__Firmicutes__c___00000204	0,68	1	1	2,42	1,41	0	0	0	0	1,713
p__Verrucomicrobia__g__Akkermansia_00000496	0,7	1	1	1,1	0,6	0	0	0	0	1,828
p__Verrucomicrobia__g__Akkermansia_00000451	0,7	1	1	1,23	0,67	0	0	0	0	1,851
p__Verrucomicrobia__g__Akkermansia_00000436	0,7	1	1	1,27	0,71	0	0	0	0	1,799
p__Verrucomicrobia__g__Akkermansia_00000394	0,7	1	1	1,37	0,82	0	0	0	0	1,672
p__Verrucomicrobia__g__Akkermansia_00000365	0,7	1	1	1,42	0,87	0	0	0	0	1,64
p__Verrucomicrobia__g__Akkermansia_00000337	0,7	1	1	1,58	0,94	0	0	0	0	1,67
p__Firmicutes__o__Lactobacillales_00000275	0,7	1	1	2,07	1,07	0	0	0	0	1,944
p__Firmicutes__g__Robinsoniella__s__peoriensis_00000306	0,7	1	1	2,09	1,12	0	0	0	0	1,867
p__Firmicutes__g__Peptococcus_00000345	0,7	1	1	1,19	0,74	0	0	0	0	1,603
p__Firmicutes__g__Moryella__s__indoligenes_00000233	0,7	1	1	2,55	1,25	0	0	0	0	2,048
p__Firmicutes__g__Faecalibaculum__s__rodentium_00000351	0,7	1	1	1,15	0,64	0	0	0	0	1,789
p__Firmicutes__g__Faecalibaculum__s__rodentium_00000312	0,7	1	1	1,21	0,78	0	0	0	0	1,546
p__Firmicutes__g__Faecalibaculum__s__rodentium_00000293	0,7	1	1	1,36	0,73	0	0	0	0	1,869
p__Firmicutes__g__Faecalibaculum__s__rodentium_00000277	0,7	1	1	1,43	0,8	0	0	0	0	1,782
p__Firmicutes__g__Faecalibaculum__s__rodentium_00000258	0,7	1	1	1,52	0,85	0	0	0	0	1,791
p__Firmicutes__g__Faecalibaculum__s__rodentium_00000254	0,7	1	1	1,55	0,85	0	0	0	0	1,823
p__Firmicutes__g__Faecalibaculum__s__rodentium_00000249	0,7	1	1	1,5	0,96	0	0	0	0	1,571
p__Firmicutes__g__Desulfotomaculum__s__sp._SN1_H_00000210	0,7	1	1	1,65	1,27	0	0	0	0	1,299
p__Firmicutes__g__Clostridium__s__sp._ASF502_00000244	0,7	1	1	1,61	0,82	0	0	0	0	1,975
p__Firmicutes__g__Clostridium_00000222	0,7	1	1	1,77	0,8	0	0	0	0	2,217
p__Firmicutes__g__Clostridium_00000205	0,7	1	1	1,96	0,8	0	0	0	0	2,455
p__Firmicutes__g__Clostridium_00000171	0,7	1	1	2,98	2,01	4,24	0	0,339216282	0	1,484
p__Firmicutes__g__Blautia__s__Ruminococcus_gnavus_00000135	0,7	1	1	5,69	5,09	6,56	0	0,524825192	0	1,117
p__Firmicutes__g__Anaerotaenia__s__torta_00000191	0,7	1	1	4	3,17	0	2,45	0	0,187805663	1,26
p__Firmicutes__g__Anaerotaenia__s__torta_00000133	0,7	1	1	4,06	2,86	0	0	0	0	1,421
p__Firmicutes__g__Lachnoclostridium__s__Clostridium_asparagiforme_00000141	0,71	1	1	6,4	4,66	6,48	3,5	0,518424884	0,268293805	1,372
p__Firmicutes__g__Lachnoclostridium__s__Clostridium_asparagiforme_00000038	0,71	1	1	12,53	9,15	13,42	7,09	1,073651535	0,543486593	1,37
p__Firmicutes__g__Clostridium__s__sp._Culture_Jar13_00000177	0,71	1	1	2,36	2,89	2,83	2,29	0,226410868	0,175540804	-1,224
p__Firmicutes__g__Clostridium__s__fusiformis_00000211	0,71	1	1	2,37	2,02	3	1,94	0,240011521	0,148711423	1,173
p__Firmicutes__o__Clostridiales_00000043	0,72	1	1	10,73	9,21	9,27	8,98	0,741635599	0,688365248	1,165
p__Firmicutes__g__Murimonas__s__intestini_00000087	0,72	1	1	7,59	8,39	7,48	11,28	0,598428725	0,864672605	-1,106
p__Firmicutes__g__Clostridium__s__sp._MarseilleP2776_00000097	0,72	1	1	4,08	4,42	4,24	4,89	0,339216282	0,374844773	-1,083
p__Firmicutes__g__Clostridium__s__sp._Clone27_00000024	0,72	1	1	9,4	7,57	9,38	7,29	0,750436021	0,558817668	1,242
p__Actinobacteria__g__Eggerthella__s__sp._YY7918_00000108	0,72	1	1	8,76	11,12	8,72	15,47	0,697633486	1,185858617	-1,27
p__Verrucomicrobia__g__Akkermansia_00000212	0,73	1	1	2,06	2,64	0	0	0	0	-1,282
p__Proteobacteria__g__Parasutterella__s__excrementihominis_00000025	0,73	1	1	14,94	12,21	13,08	12,34	1,04645023	0,9459273	1,224
p__Bacteroidetes__g__Alistipes__s__massiliensis_00000010	0,73	1	1	29,23	29,85	25,38	32,36	2,030497464	2,480567863	-1,021
p__Firmicutes__g__Oscillibacter__s__valericigenes_00000226	0,74	1	1	2,56	1,82	0	0	0	0	1,403
p__Verrucomicrobia__g__Akkermansia_00000412	0,77	1	1	1,29	0,8	0	0	0	0	1,614
p__Verrucomicrobia__g__Akkermansia_00000385	0,77	1	1	1,35	0,87	0	0	0	0	1,556
p__Firmicutes__o__Clostridiales_00000063	0,77	1	1	5,51	8,5	6,71	7,13	0,536825768	0,546552808	-1,543
p__Firmicutes__g__Clostridium__s__sp._Clone27_00000019	0,77	1	1	10,36	8,41	10,25	7,75	0,820039362	0,594079139	1,232
p__Firmicutes__g__Clostridium__s__sp._A9_00000372	0,77	1	1	1,03	0,62	0	0	0	0	1,65
p__Firmicutes__g__Clostridium_00000014	0,77	1	1	11,27	8,85	7,28	6,62	0,582427957	0,507458568	1,272
p__Firmicutes__g__Candidatus_Stoquefichus_00000119	0,77	1	1	4,01	4,19	4,47	5,2	0,357617166	0,398607938	-1,044
p__Verrucomicrobia__g__Akkermansia_00000366	0,79	1	1	1,24	1,5	0	0	0	0	-1,211
p__Verrucomicrobia__g__Akkermansia_00000294	0,79	1	1	1,5	1,85	0	0	0	0	-1,229
p__Verrucomicrobia__g__Akkermansia_00000229	0,81	1	1	2,53	2,15	0	0	0	0	1,176
p__Verrucomicrobia__g__Akkermansia_00000216	0,81	1	1	2,67	2,37	0	0	0	0	1,131
p__Verrucomicrobia__g__Akkermansia_00000146	0,81	1	1	4,06	3,26	0	0	0	0	1,243
p__Verrucomicrobia__g__Akkermansia_00000109	0,81	1	1	4,62	4,08	0	0	0	0	1,132
p__Firmicutes__g__Tyzzerella__s__sp._MarseilleP3062_00000101	0,81	1	1	5,85	4,31	0	0	0	0	1,356
p__Firmicutes__g__Pseudoflavonifractor__s__sp._MarseilleP3106_00000190	0,81	1	1	2,87	2,24	0	0	0	0	1,277
p__Firmicutes__g__Clostridium__s__sp._Clone44_00000195	0,81	1	1	2,2	1,49	0	0	0	0	1,479
p__Firmicutes__g__Blautia__s__Ruminococcus_gnavus_00000091	0,81	1	1	6,98	5,89	0	0	0	0	1,184
k__Unclassified_00000280	0,81	1	1	2,2	1,66	0	0	0	0	1,32
p__Firmicutes__g__Lactobacillus__s__reuteri_00000105	0,82	1	1	3,37	3,97	0	1,87	0	0,143345547	-1,178
p__Firmicutes__g__Desulfitobacterium_00000200	0,82	1	1	2,39	2,13	0	1,66	0	0,127247919	1,126
p__Verrucomicrobia__g__Akkermansia_00000075	0,83	1	1	6,05	6,93	5,39	6,13	0,431220699	0,469897435	-1,145
p__Firmicutes__o__Clostridiales_00000159	0,83	1	1	4,09	3,87	5,57	3	0,44562139	0,229966118	1,056

p__Firmicutes__g__Oscillibacter__s__ruminantium_00000130	0,83	1	1	4,52	5,19	5,29	6,56	0,423220315	0,502859245	-1,149
p__Firmicutes__g__Eubacterium__s__desmolans_00000045	0,83	1	1	6,26	8,38	6,78	7,97	0,542426036	0,610943321	-1,338
p__Firmicutes__g__Clostridium__s__sp._Culture41_00000039	0,83	1	1	8,3	8,67	8,43	9	0,674432373	0,689898355	-1,046
p__Firmicutes__g__Clostridium__s__sp._BSY12_00000020	0,83	1	1	6,69	8,42	6,71	6,03	0,536825768	0,462231898	-1,259
p__Firmicutes__g__Anaerotruncus__s__sp._MarseilleP2769_00000035	0,83	1	1	13,89	13,29	14,93	10,75	1,194457334	0,824045257	1,045
p__Verrucomicrobia__g__Akkermansia_00000001	0,84	1	1	36,28	40,34	28,43	34,33	2,274509176	2,631578947	-1,112
p__Firmicutes__g__Ruminiclostridium__s__Clostridium_viride_00000031	0,84	1	1	17,54	17,25	19,31	18,42	1,544874154	1,411991967	1,017
p__Firmicutes__g__Faecalibaculum__s__rodentium_00000006	0,84	1	1	14,58	15,03	11,79	12,13	0,943245276	0,929829672	-1,031
p__Firmicutes__g__Clostridium__s__sp._ASF502_00000011	0,84	1	1	13,59	16,45	13,75	15,18	1,100052803	1,163628559	-1,211
p__Actinobacteria__g__Eggerthella__s__sp._YY7918_00000070	0,84	1	1	13,76	16	12,25	13,34	0,980047042	1,022582673	-1,162
p__Verrucomicrobia__g__Akkermansia_00000432	0,85	1	1	1,23	0,78	0	0	0	0	1,568
p__Firmicutes__g__Ruminococcus__s__sp._MarseilleP3217_00000424	0,85	1	1	1,57	1,09	0	0	0	0	1,432
p__Firmicutes__g__Ruminococcus__s__bicirculans_00000321	0,85	1	1	1,87	1,57	0	0	0	0	1,192
p__Firmicutes__g__Lachnoclostridium_00000357	0,85	1	1	1,58	1,2	0	0	0	0	1,311
p__Firmicutes__g__Lachnoclostridium_00000278	0,85	1	1	2,14	1,42	0	0	0	0	1,51
p__Firmicutes__g__Clostridium__s__sp._Clone27_00000163	0,85	1	1	1,52	1,96	0	0	0	0	-1,285
p__Firmicutes__g__Clostridium__s__sp._AT5_00000389	0,85	1	1	0,94	0,64	0	0	0	0	1,464
p__Firmicutes__g__Clostridium__s__sp._AT5_00000247	0,85	1	1	1,32	1,09	0	0	0	0	1,211
p__Verrucomicrobia__g__Akkermansia_00000302	0,86	1	1	1,49	1,79	0	0	0	0	-1,202
p__Verrucomicrobia__g__Akkermansia_00000283	0,87	1	1	2,08	1,76	0	0	0	0	1,184
p__Firmicutes__g__Eubacterium__s__sp._SR79_00000265	0,87	1	1	1,52	2,02	0	1,94	0	0,148711423	-1,332
p__Firmicutes__g__Pseudoflavonifractor__s__sp._MarseilleP3106_00000209	0,88	1	1	3,02	2,63	4	2,12	0,320015361	0,16250939	1,15
p__Firmicutes__g__Faecalibaculum__s__rodentium_00000129	0,88	1	1	3,35	3,15	3,46	1,66	0,276813287	0,127247919	1,065
p__Firmicutes__g__Candidatus_Stoquefichus_00000201	0,88	1	1	2,16	2,63	0	3,38	0	0,25909516	-1,221
p__Firmicutes__g__Clostridium__s__sp._MarseilleP2776_00000057	0,89	1	1	7	6,81	6,56	6,67	0,524825192	0,511291336	1,028
p__Firmicutes__g__Clostridium__s__sp._AT5_00000008	0,89	1	1	14,99	13,27	14,8	13,24	1,184056835	1,014917136	1,13
p__Actinobacteria__g__Asaccharobacter__s__sp._WCA131CoC2_00000151	0,89	1	1	9,42	8,63	8,89	9,16	0,711234139	0,702163215	1,091
p__Verrucomicrobia__g__Akkermansia_00000505	0,91	1	1	0,64	0,78	0	0	0	0	-1,224
p__Verrucomicrobia__g__Akkermansia_00000439	0,91	1	1	0,74	0,91	0	0	0	0	-1,229
p___Unclassified.Bacteria__c___00000300	0,91	1	1	1,32	1,55	0	0	0	0	-1,174
p__Nematoda__g__Haemonchus__s__placei_00000413	0,91	1	1	1,51	1,82	0	0	0	0	-1,203
p__Firmicutes__g__Ruminococcus__s__sp._MarseilleP3217_00000399	0,91	1	1	0,9	1,45	0	0	0	0	-1,61
p__Firmicutes__g__Ruminococcus__s__sp._00000316	0,91	1	1	1,23	1,73	0	0	0	0	-1,409
p__Firmicutes__g__Ruminiclostridium__s__sp._KB18_00000484	0,91	1	1	0,88	1,17	0	0	0	0	-1,326
p__Firmicutes__g__Lachnoclostridium__s__Clostridium_saccharolyticum_00000125	0,91	1	1	2,59	3,6	0	0	0	0	-1,389
p__Firmicutes__g__Lachnoclostridium_00000417	0,91	1	1	0,86	1,19	0	0	0	0	-1,388
p__Firmicutes__g__Lachnoclostridium_00000393	0,91	1	1	0,81	1,33	0	0	0	0	-1,649
p__Firmicutes__g__Lachnoclostridium_00000379	0,91	1	1	0,83	1,36	0	0	0	0	-1,639
p__Firmicutes__g__Herbinix_00000182	0,91	1	1	2,01	2,54	0	0	0	0	-1,263
p__Firmicutes__g__Dorea__s__formicigenerans_00000315	0,91	1	1	1,74	2,17	0	0	0	0	-1,246
p__Firmicutes__g__Clostridium__s__sp._MK12_00000384	0,91	1	1	0,47	0,85	0	0	0	0	-1,792
p__Firmicutes__g__Clostridium__s__sp._Culture41_00000388	0,91	1	1	0,57	0,74	0	0	0	0	-1,304
p__Firmicutes__g__Clostridium__s__sp._AT5_00000214	0,91	1	1	1,02	1,33	0	0	0	0	-1,307
p__Firmicutes__g__Clostridium_00000086	0,91	1	1	1,93	2,62	0	0	0	0	-1,358
p__Firmicutes__f__Ruminococcaceae_00000440	0,91	1	1	1,05	1,24	0	0	0	0	-1,178
p__Firmicutes__f__Ruminococcaceae_00000421	0,91	1	1	1,06	1,3	0	0	0	0	-1,228
p__Bacteroidetes__g__Alistipes_00000477	0,91	1	1	0,95	1,31	0	0	0	0	-1,385
p__Verrucomicrobia__g__Akkermansia_00000395	0,93	1	1	1,16	1,4	0	0	0	0	-1,213
p__Verrucomicrobia__g__Akkermansia_00000369	0,93	1	1	1,24	1,46	0	0	0	0	-1,177
p__Verrucomicrobia__g__Akkermansia_00000314	0,93	1	1	1,53	1,66	0	0	0	0	-1,087
p__Verrucomicrobia__g__Akkermansia_00000309	0,93	1	1	1,5	1,76	0	0	0	0	-1,173
p__Verrucomicrobia__g__Akkermansia_00000307	0,93	1	1	1,65	1,58	0	0	0	0	1,042
p__Verrucomicrobia__g__Akkermansia_00000296	0,93	1	1	1,56	1,75	0	0	0	0	-1,122
p__Verrucomicrobia__g__Akkermansia_00000286	0,93	1	1	1,61	1,78	0	0	0	0	-1,105
p__Verrucomicrobia__g__Akkermansia_00000282	0,93	1	1	1,73	1,74	0	0	0	0	-1,007
p__Verrucomicrobia__g__Akkermansia_00000236	0,93	1	1	1,95	2,15	0	0	0	0	-1,1
p__Verrucomicrobia__g__Akkermansia_00000220	0,93	1	1	2,1	2,43	0	0	0	0	-1,153
p__Verrucomicrobia__g__Akkermansia_00000217	0,93	1	1	2,28	2,19	0	0	0	0	1,045
p__Verrucomicrobia__g__Akkermansia_00000187	0,93	1	1	2,67	2,5	0	0	0	0	1,071
p__Verrucomicrobia__g__Akkermansia_00000186	0,93	1	1	2,48	2,82	0	0	0	0	-1,139
p__Firmicutes__o__Clostridiales_00000103	0,93	1	1	3,24	3,83	0	0	0	0	-1,181
p__Firmicutes__g__Ruminococcus__s__bicirculans_00000273	0,93	1	1	2,93	2,34	0	0	0	0	1,252
p__Firmicutes__g__Clostridium__s__sp._Culture57_00000121	0,93	1	1	3,09	1,65	0	0	0	0	1,876
p__Firmicutes__g__Clostridium__s__sp._Clone40_00000029	0,93	1	1	3,78	5,6	0	0	0	0	-1,482
p__Firmicutes__g__Clostridium__s__sp._BNL1100_00000270	0,93	1	1	1,23	1,35	0	0	0	0	-1,096
p__Firmicutes__g__Blautia__s__Ruminococcus_gnavus_00000243	0,93	1	1	2,26	3,16	0	0	0	0	-1,397
p__Verrucomicrobia__g__Akkermansia_00000213	0,94	1	1	2,45	2,64	0	0	0	0	-1,078
p__Verrucomicrobia__g__Akkermansia_00000207	0,94	1	1	2,84	2,41	0	0	0	0	1,177
p___Unclassified.Bacteria__c___00000046	0,94	1	1	10,55	10,43	7,55	9,3	0,604028993	0,712894967	1,012
p__Firmicutes__o__Clostridiales_00000166	0,94	1	1	3,1	2,89	0	0	0	0	1,072
p__Firmicutes__g__Ruminococcus_00000093	0,94	1	1	8,42	6,16	7,62	5,47	0,609629262	0,419304889	1,367
p__Firmicutes__g__Oscillospira__s__guilliermondii_00000178	0,94	1	1	4,08	4,76	5,48	6,43	0,438421044	0,492894047	-1,166
p__Firmicutes__g__Holdemanella__s__biformis_00000083	0,94	1	1	4,92	5,5	5,1	5,34	0,408019585	0,409339691	-1,118
p__Firmicutes__g__Coprococcus__s__catus_00000111	0,94	1	1	9,5	9,77	10	10,15	0,800038402	0,778052034	-1,028
p__Firmicutes__g__Clostridium_00000266	0,94	1	1	1,49	1,43	0	0	0	0	1,042
p__Firmicutes__g__Lachnoclostridium_00000007	0,95	1	1	24,12	23,24	20,2	15,57	1,616077572	1,193524154	1,038
p__Actinobacteria__g__Paraeggerthella__s__hongkongensis_00000099	0,95	1	1	12,61	12,81	11,09	12,59	0,887242588	0,965091143	-1,016
p__Actinobacteria__g__Enterorhabdus__s__mucosicola_00000034	0,95	1	1	21,14	20,67	19,05	20,78	1,524073156	1,592898646	1,023
p__Verrucomicrobia__g__Akkermansia_00000539	1	1	1	0,64	0,67	0	0	0	0	-1,044
p__Verrucomicrobia__g__Akkermansia_00000435	1	1	1	0,78	0,88	0	0	0	0	-1,126
p__Verrucomicrobia__g__Akkermansia_00000360	1	1	1	1,32	1,45	0	0	0	0	-1,097
p__Verrucomicrobia__g__Akkermansia_00000343	1	1	1	1,38	1,5	0	0	0	0	-1,086
p__Verrucomicrobia__g__Akkermansia_00000342	1	1	1	0,97	1,08	0	0	0	0	-1,115
p__Verrucomicrobia__g__Akkermansia_00000333	1	1	1	1,47	1,54	0	0	0	0	-1,048
p__Verrucomicrobia__g__Akkermansia_00000323	1	1	1	1,53	1,56	0	0	0	0	-1,018
p__Verrucomicrobia__g__Akkermansia_00000322	1	1	1	1,51	1,58	0	0	0	0	-1,051
p__Verrucomicrobia__g__Akkermansia_00000318	1	1	1	1,6	1,48	0	0	0	0	1,083
p__Verrucomicrobia__g__Akkermansia_00000179	1	1	1	2,65	2,72	0	0	0	0	-1,024
p__Verrucomicrobia__g__Akkermansia_00000172	1	1	1	3,35	3,07	0	0	0	0	1,089
p__Verrucomicrobia__g__Akkermansia_00000168	1	1	1	3,45	3,16	0	0	0	0	1,094
p__Verrucomicrobia__g__Akkermansia_00000053	1	1	1	7,55	7,24	5,39	6,38	0,431220699	0,489061278	1,042
p__Proteobacteria__g__Helicobacter__s__hepaticus_00000267	1	1	1	2,56	2,39	0	0	0	0	1,074
p__Firmicutes__o__Lactobacillales_00000422	1	1	1	0,94	0,82	0	0	0	0	1,147
p__Firmicutes__o__Lactobacillales_00000225	1	1	1	2,25	2,28	0	0	0	0	-1,013
p__Firmicutes__o__Clostridiales_00000444	1	1	1	0,7	0,78	0	0	0	0	-1,117
p__Firmicutes__o__Clostridiales_00000131	1	1	1	2,85	1,32	0	0	0	0	2,15
p__Firmicutes__o__Clostridiales_00000094	1	1	1	5,12	4,55	4,69	4,61	0,37521801	0,353381268	1,124
p__Firmicutes__g__Turicibacter_00000398	1	1	1	0,73	0,58	0	0	0	0	1,263
p__Firmicutes__g__Ruminococcus_00000162	1	1	1	3,15	2,37	0	0	0	0	1,33
p__Firmicutes__g__Ruminiclostridium__s__Clostridium_cellobioparum_00000405	1	1	1	1,15	1,29	0	0	0	0	-1,122
p__Firmicutes__g__Roseburia__s__sp._MC_37_00000145	1	1	1	5,49	5,66	5,66	3,57	0,452821735	0,273659681	-1,03
p__Firmicutes__g__Pseudoflavonifractor__s__sp._MarseilleP3106_00000185	1	1	1	2,83	3,73	0	4,95	0	0,379444095	-1,317
p__Firmicutes__g__Moryella__s__indoligenes_00000153	1	1	1	4,4	4,27	0	2,74	0	0,210035721	1,031
p__Firmicutes__g__Marvinbryantia__s__formatexigens_00000262	1	1	1	1,61	1,46	0	0	0	0	1,101
p__Firmicutes__g__Lactobacillus_00000049	1	1	1	4,38	0,83	0	0	0	0	5,251
p__Firmicutes__g__Lachnoclostridium__s__Clostridium_saccharolyticum_00000082	1	1	1	8,49	7,57	7,07	8,67	0,56562715	0,664602082	1,122
p__Firmicutes__g__Erysipelatoclostridium__s__Clostridium_cocleatum_00000077	1	1	1	3,82	4,71	3,32	2,12	0,265612749	0,16250939	-1,233
p__Firmicutes__g__Coprococcus__s__catus_00000147	1	1	1	9,45	9	10,63	10,65	0,850440821	0,81637972	1,05
p__Firmicutes__g__Clostridium__s__sp._MarseilleP2776_00000041	1	1	1	7,27	8,19	8,72	8,04	0,697633486	0,616309197	-1,126
p__Firmicutes__g__Clostridium__s__sp._Culture41_00000180	1	1	1	3,03	3,28	3,74	3,74	0,299214362	0,286691094	-1,083
p__Firmicutes__g__Clostridium__s__sp._Culture27_00000089	1	1	1	3,06	4,48	4,24	1,94	0,339216282	0,148711423	-1,463
p__Firmicutes__g__Clostridium__s__sp._Culture1_00000319	1	1	1	0,86	0,69	0	0	0	0	1,248
p__Firmicutes__g__Clostridium__s__sp._Culture1_00000136	1	1	1	3,11	3,24	4,12	2,55	0,329615822	0,195471201	-1,041
p__Firmicutes__g__Clostridium__s__sp._Clone49_00000085	1	1	1	4,03	4,27	3,74	3,54	0,299214362	0,27136002	-1,06
p__Firmicutes__g__Clostridium__s__sp._Clone27_00000016	1	1	1	10,65	10,23	9,9	8,81	0,792038018	0,675333834	1,041
p__Firmicutes__g__Clostridium__s__sp._Clone17_00000371	1	1	1	0,65	0,71	0	0	0	0	-1,08
p__Firmicutes__g__Clostridium__s__sp._Clone17_00000096	1	1	1	3,46	4,1	4,24	2,23	0,339216282	0,170941481	-1,183
p__Firmicutes__g__Clostridium__s__sp._BNL1100_00000339	1	1	1	0,74	0,73	0	0	0	0	1,022
p__Firmicutes__g__Clostridium__s__sp._AT5_00000386	1	1	1	0,65	0,67	0	0	0	0	-1,019
p__Firmicutes__g__Clostridium__s__sp._AT5_00000276	1	1	1	1,31	1,24	0	0	0	0	1,055
p__Firmicutes__g__Clostridium__s__sp._ASF502_00000071	1	1	1	4,01	4,56	2,65	1,8	0,212010176	0,137979671	-1,137
p__Firmicutes__g__Clostridium__s__sp._A9_00000170	1	1	1	1,7	1,14	0	0	0	0	1,483

p__Firmicutes__g__Clostridium__s__fusiformis_00000253	1	1	1	1,14	0,69	0	0	0	0	1,664
p__Firmicutes__g__Clostridium__s__fusiformis_00000033	1	1	1	8,91	8,98	8,12	9,93	0,649631182	0,761187852	-1,008
p__Firmicutes__g__Clostridium_00000289	1	1	1	1	0,58	0	0	0	0	1,734
p__Firmicutes__g__Clostridium_00000271	1	1	1	1,12	0,53	0	0	0	0	2,134
p__Firmicutes__g__Clostridium_00000260	1	1	1	1,12	0,67	0	0	0	0	1,686
p__Firmicutes__g__Blautia__s__Ruminococcus_gnavus_00000313	1	1	1	1,54	1,43	0	0	0	0	1,073
p__Bacteroidetes__g__Alistipes__s__massiliensis_00000463	1	1	1	1,25	1	0	0	0	0	1,246
p__Bacteroidetes__g__Alistipes__s__massiliensis_00000367	1	1	1	1,36	1,51	0	0	0	0	-1,114
p__Bacteroidetes__g__Alistipes__s__massiliensis_00000305	1	1	1	1,78	1,61	0	0	0	0	1,107
k__Unclassified_00000437	1	1	1	0,9	0,78	0	0	0	0	1,155
